# Prenatal manifestation and management of a mother and child affected by spondyloperipheral dysplasia with a C-propeptide mutation in COL2A1: case report

**DOI:** 10.1186/1750-1172-6-7

**Published:** 2011-02-28

**Authors:** Maria Francesca Bedeschi, Vera Bianchi, Barbara Gentilin, Lorenzo Colombo, Federica Natacci, Sabrina Giglio, Elena Andreucci, Laura Trespidi, Barbara Acaia, Andrea Superti Furga, Faustina Lalatta

**Affiliations:** 1 U.O.D. Genetica Medica, Dipartimento Salute della donna, del bambino, del neonato Fondazione IRCCS Policlinico, Mangiagalli e Regina Elena, Milano, Italia; 2 Unità di terapia intensiva neonatale, Fondazione IRCCS, Ospedale Maggiore Policlinico, Mangiagalli e Regina Elena, Milano, Italia; 3 Unità di Genetica Medica, Azienda Ospedaliera Universitaria Meyer, Firenze, Italia; 4 I Clinica Ginecologica e Ostetrica, Fondazione IRCCS Policlinico, Mangiagalli e Regina Elena, Milano, Italia; 5 Dipartimento di Pediatria, Università di Friburgo, Germania

## Abstract

It is not unusual for patients with "rare" conditions, such as skeletal dysplasias, to remain undiagnosed until adulthood. In such cases, a pregnancy may unexpectedly reveal hidden problems and special needs. A 28 year old primigravida was referred to us at 17 weeks for counselling with an undiagnosed skeletal dysplasia with specific skeletal anomalies suggesting the collagen 2 disorder, spondyloperipheral dysplasia (SPD; MIM 156550).

She was counselled about the probability of dominant inheritance and was offered a prenatal diagnosis by sonography. US examination at 17, 18 and 20 weeks revealed fetal macrocephaly, a narrow thorax, and shortening and bowing of long bones. The parents elected to continue the pregnancy. At birth the baby showed severe respiratory distress for four weeks which then resolved. Mutation analysis of both mother and child revealed a hitherto undescribed heterozygous nonsense mutation in the C-propeptide coding region of COL2A1 confirming the diagnosis of SPD while reinforcing the genotype-phenotype correlations between C-propeptide COL2A1 mutations and the SPD-Torrance spectrum. This case demonstrates the importance of a correct diagnosis even in adulthood, enabling individuals affected by rare conditions to be made aware about recurrence and pregnancy-associated risks, and potential complications in the newborn.

## Background

Type II collagenopathies range from lethal forms (Achondrogenesis II, Hypochondrogenesis), to severe diseases (spondyloepiphyseal dysplasia congenital, Kniest dysplasia) to mild phenotypes (Stickler dysplasia, a mild dominant spondyloarthropathy) [1]. Heterozygous mutations mapping to different parts of the triple helical domain of the COL2A1 gene have been identified for most of these disorders. Spondyloperipheral dysplasia (SPD-OMIM 156550) is a skeletal dysplasia with platyspondyly and brachydactyly E changes, first described in 1977 [2]. Additional cases were later reported with some similarities but with considerable clinical variability [3-5] but all of them exhibited autosomal dominant inheritance with duplications, truncating mutations and missense mutations of C-propeptide of the COL2A1 gene having been previously reported [4-6]. Here we describe a family, a mother and her son, with SPD and a novel truncating mutation in the C-propeptide domain of COL2A1.

## Case presentation: Patient 1

Patient 1, the mother, is the first girl of healthy, non-consanguineous Caucasian parents who was spontaneously delivered at term after an uneventful pregnancy. Family history was unremarkable for mental retardation or birth defects.

Her growth parameters were always below the 3rd centile and she reached her final height of 115 cm at 16 years old. At 2 months she underwent surgical correction of club feet.

At 15 years old a diagnosis of Dyggve-Melchior-Clausen (DMC) syndrome was made in another Centre and the parents were advised accordingly. She was diagnosed with bilateral otosclerosis at 16 years and underwent a tympanum replacement in the left ear. She has had mild conductive hypoacusia since then. She developed moderate scoliosis and suffered intermittent back pain and hip and knee stiffness. Despite these medical problems, she was healthy and she had an active life.

At time of her first pregnancy she was referred to our Centre at 17 weeks of gestation following a sonogram which had revealed a fetal femur length below the 3rd centile.

**Figure 1:**
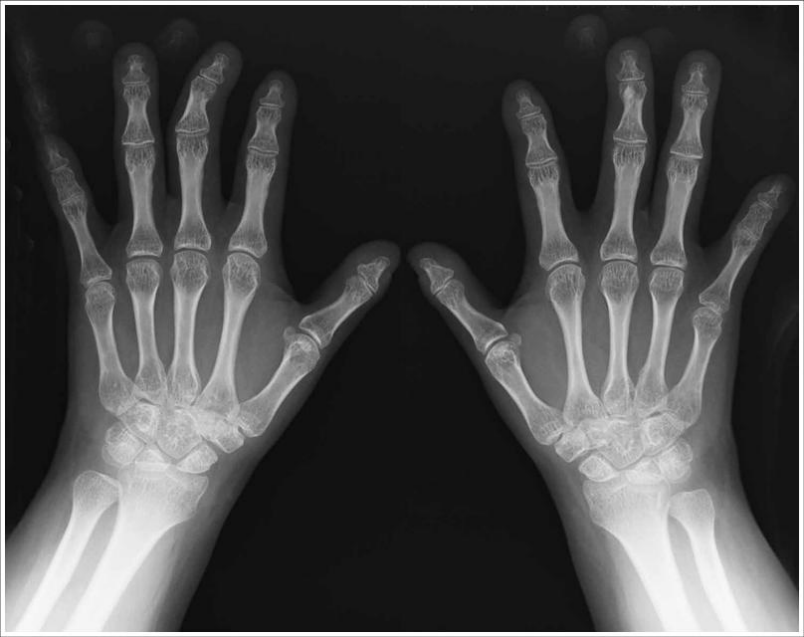
**Radiographs of hands**. Note mild shortening of the fifth metacarpal of the hands.

**Figure 2:**
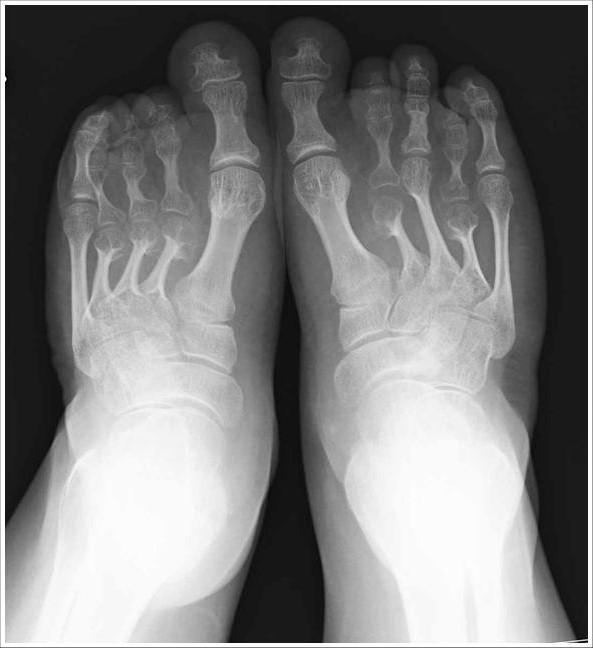
**Radiographs of feet**. Note the 2^nd ^and 4^th ^metatarsal bones and the great toe.

At the time of referral her auxological parameters measured: weight 55 kg (25-50th centile), height 115 cm (-4DS), OFC 57 cm (97th centile). Arm length was 121 cm, lower segment length was 60 cm and upper segment length 55 cm. Hand length was 15 cm (<3rd centile) and middle finger 5,5 cm (<3rd centile). She was macrocephalic, with a round face and mild midface hypoplasia. Present were kyphosis, mild lumbar lordosis and bilateral E brachydactyly of the hands and shortened toes 2 to 5. A prominent first toe (Brachydactyly E) was also noted (Figure 1, 2).

**Figure 3:**
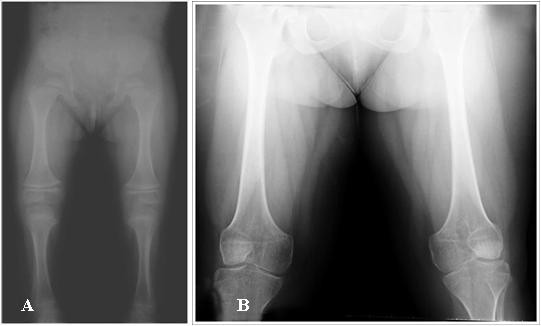
**Radiographs of patient 1**. **A**. 4 years. **B**. 28 years. Note short iliac bones, flattened femoral heads, short and broad femoral neck. Short femoral and tibial diaphysys, methaphyseal widening and broad femoral ends.

**Figure 4:**
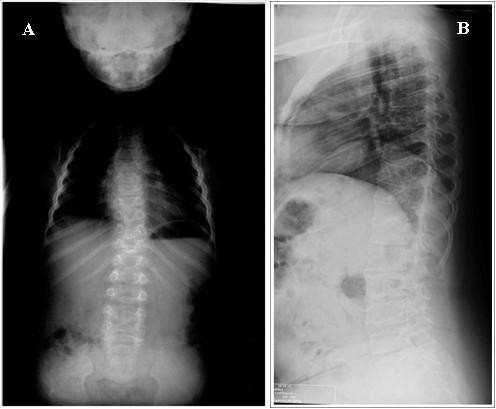
**Radiographs of patient 1**. **A**. 6 years. **B**. 28 years. Note generalized platyspondyly, thoracolumbar scoliosis.

A re-evaluation of the radiographic images taken at ages 4 and 28 years, demonstrated platyspondyly, reduced intervertebral space, bell shaped thorax, scoliosis, broad ilia with hypoplasia of the basilar portions, shortening of the long bones with flared ends (methaphyseal enlargement), short femoral ends, mild genu valgum, bilateral brachymetaphalangia of the fifth finger of the hand (Brachydactyly E-like) and brachymetatarsia of the four toes similar to brachydactyly E (Figure 3, 4).

Except for the presence of the brachydactyly, many of the patient's clinical and radiographic features were compatible with a type II collagen disorder, in particular with spondyloepiphyseal dysplasia congenita or Kniest dysplasia. Specifically, our patient demonstrated a number of similarities with the cases described by Zankl et al 2004 [4] which had been used to define SPD.

**Figure 5:**
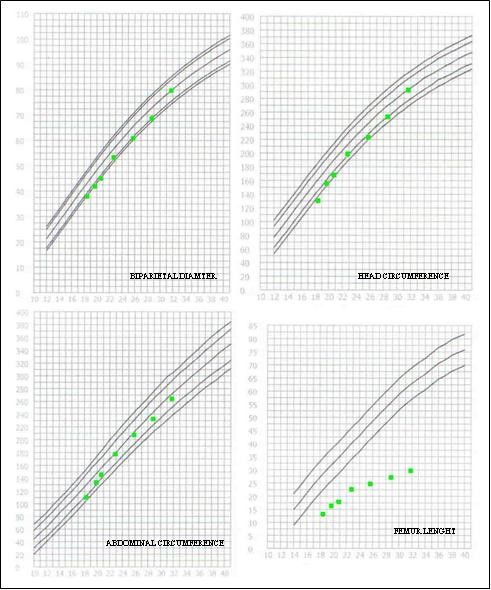
**Fetal auxological measurements showing the shortening of the femur beginning with the 15th the week of gestation**.

The woman was informed about the high risk of transmission and an ultrasound prenatal diagnosis was offered. Ultrasound examinations between 18 and 32 week of gestation revealed fetal macrocephaly, narrowed thorax, severe shortening and bowing of long bones (<<3° centile), a cardiac-thoracic ratio of 0,48 with a cardiothoracic/cardio abdominal ratio of 80%, lateral cerebral ventricles measuring 7 mm, regular posterior cranial fossa, and normal amniotic fluid volume (Figure 5). Following extensive counseling the parents decided to continue the pregnancy.

During the pregnancy, the woman was offered a complete workup including a cardiac, pneumological, orthopaedic and obstetric examinations every month from the 17th to the 32nd week. All parameters remained within the normal range.

She reported a reasonably good quality of life during the second trimester but in the 32nd week she was admitted to the hospital to induce foetal lung maturation by corticosteroids.

The baby, a male, was born through a transverse caesarean section performed at the 33rd week under spinal anesthesia.

## Case presentation: Patient 2

Patient 2, the child had the following measurements at birth: weight 1780 gr (10-25th centile), length 32 cm (-4 DS) and OFD 32 cm (75th centile). Apgar scores were 3, 7 and 8 at one, five and ten minutes respectively. Because of difficulties with respiration a naso-tracheal tube was inserted and breathing was aided by mechanical ventilation. The child was initially treated with a high-frequency ventilation for one day and with conventional mechanical ventilation for 12 days. For the following 25 days the child was maintained in nasal Continuous Positive Airway Pressure (CPAP). Oxygen therapy was discontinued at 41 weeks of age. A single dose of exogenous surfactant was administered. Afterwards occasional desaturation episodes were noted with spontaneous resolution. Enteric feeding was initiated on the second day of life and was maintained until week 38 when the baby was started on bottle feeding.

**Figure 6:**
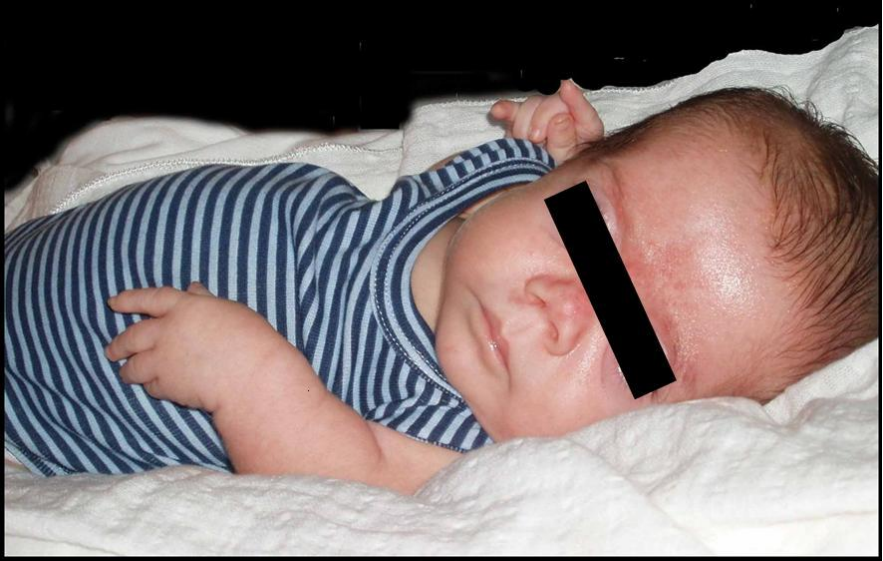
**Patient 2, 2 years**. Note flat mid-face, depressed nasal bridge.

Clinical evaluation showed a flat face, apparent hypertelorism, depressed nasal bridge, small upturned nose, midface hypoplasia, micrognathia, ulnar deviation of hands and club feet (Figure 6).

**Figure 7:**
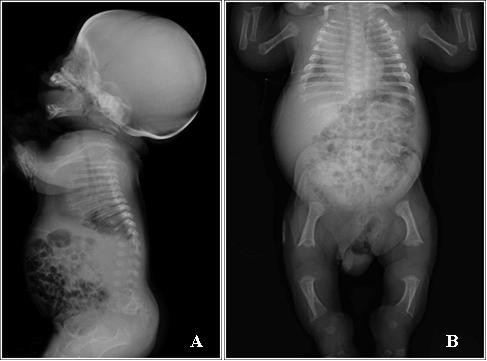
**Radiological features of patient 2**. There is retarded ossification of the skeleton with absence of the ossification centres of pubic bones and distal femoral and proximal tibial epiphyses. Mild platyspondyly with ovoid-pear-shaped vertebral bodies. The tubular bones are short and broad with splayed ends and cupped metaphyses and mild bowing of the tibiae.

Cardiac examination was normal. Retinal immaturity was observed. Radiographic examination performed at 2 months of age revealed a large skull, mild platyspondyly with ovoid- pear-shaped vertebral bodies, delayed ossification of pubic bones, distal femoral and proximal tibial epiphyses, shortened and broad tubular bones with splayed and cupped metaphyses, mild bowing of the tibiae (Figure 7).

## Molecular studies

After informed consent was obtained from both individuals, blood was drawn and genomic DNA of both mother and son was extracted from peripheral blood leukocytes using standard protocols.

Coding regions, intron-exon boundaries and flanking intronic sequences of the COL2A1 gene were amplified by PCR. Amplified PCR products were monitored on 1.5% agarose gels.

PCR products were subjected to a denaturing step of 2 minutes at 96°C followed by gradual annealing. The annealing time of each cycle was 30 seconds followed by 50 cycles of cooling from 96°C to 46°C with a decremented gradient temperature of 1°C then 7 minutes at room temperature, to promote heteroduplex formation.

dHPLC analysis was performed using the Wave MD4000 Transgenomic^® ^device and analysis of the results was carried out by Navigator^® ^version 1.6.4 software.

Fragments which indicated differences from a known negative control, were sequenced bidirectionally on an ABI 3100 Genetic Analyzer (Applied Biosystems).

Each mutation was confirmed by re-amplification of a second DNA product and re-sequencing.

In patients 1 and 2 we identified a heterozygous nonsense mutation (c.4339 A > T) in exon 54, which resulted in a premature stop codon at amino acid 1447 (K1447X). (Exon and nucleotide numbering based on RefSeq NM_001844, starting at the ATG translation initiation codon).

## Discussion

This study describes a mother and her son, affected by severe dwarfism, peculiar facial features, similar radiological findings including shortened tubular bones, specific vertebral alterations being pear-shaped in infancy and platyspondylic by adulthood; metaphyseal and epiphyseal alterations and brachydactyly E. Even though our patient presented with the typical radiological features of type 2 collagenopathy, she was not correctly diagnosed until age 28 years.

This misidentification induced a series of consequences ranging from an incorrect knowledge of reproductive risk, a poor understanding of the clinical variability of her condition and a failure to correctly identify fetal and neonatal risks. After being correctly diagnosed at the 17th week of gestation this young woman was faced with the decision either to terminate or to carry on the pregnancy and face the birth of an affected child. We believe the final decision to continue the pregnancy was supported by her good self image despite the physical diversity.

Because she decided to not to terminate it was necessary to assemble a multidisciplinary team including obstetricians, orthopaedists, cardiologists, a pneumologist and an anaesthetist to monitor cardio-respiratory and orthopaedic complications. Fortunately, the mother demonstrated a good cardio-respiratory response despite her severe dwarfism and skeletal abnormalities.

Mutation analysis of our cases revealed a hitherto undescribed, heterozygous nonsense mutation in the C-propeptide coding region of COL2A1 (c.4339 A > T, K1447X) in exon 54. Mutations of the C-propeptide of collagen II are a well established cause of type II collagenopathy associated with brachydactyly E; indeed, additional cases of SPD associated to analogous mutations of C propeptide domain of COL2A1 have been previously reported [4,5].

Brachydactyly E associated with metaphyseal changes is the most peculiar clinical feature of C propeptide mutations of collagen type 2, which differentiate them from other types of collagenopathies. It must be remembered that brachydactyly E is not only associated with SPD, but is part of clinical picture of other type 2 collagenopathies such as Czech dysplasia and platyspondylic lethal skeletal dysplasia, Torrance type (PLSD-T).

Czech dysplasia is characterized by early-onset, progressive pseudo-rheumatoid arthritis, hearing loss starting in early adulthood, vertebral anomalies (mild platyspondyly, irregular plates reduced intervertebral distances), normal stature and short third and fourth toes. A specific typical missense mutation (R275C) in the triple helical domain of the COL2A1 gene has been identified [6]. In contrast to Czech dysplasia, spondyloperipheral dysplasia (SPD) is characterized by short stature, clubfeet, platyspondyly, midface hypoplasia, myopia and epiphyseal dysplasia.

Platyspondylic lethal skeletal dysplasia, Torrance type (PLSD-T) is a rare form of skeletal dysplasia characterized by platyspondyly, brachydactyly and methapyseal changes. Mutations in COL2A1 have been identified in the Torrance type [7]. While most reports of PLSD-T describe sporadic and perinatally lethal cases, patients with longer survival have been recently reported [8]. Zankl et al. reported 8 further cases of PLSD- T and found that all had COL2A1 mutations in the C-propeptide domain. The mutational spectrum included missense, stop codon and frameshift mutations. Two of those patients, who showed the rare adult form of PLSD-T, had given birth to children with a lethal PLSD-T[9].

Our patient 2 showed radiological features as platyspondyly, ovoid vertebrae, delayed ossification of pubic bones, shortened and broad tubular bones with splayed and ragged metaphyses similar to a form of PLSD-T, whereas clinical and radiological features of our patient 1 overlap with SPD. This observation further supports the conclusion that SPD and PLSD-T are not two etiologically distinct entities but belong to the same continuum phenotypic spectrum.

The aetiology of the two conditions has been recently established through the findings of mutations in C-propeptide domain of COL2A1. Of note is the characterisation of the mutation in our case, a novel nonsense mutation (c.4339 A > T) in exon 54, which resulted in a premature stop codon at amino acid 1447 (K1447X). This mutation is located at the farthest 3'end of the domain analogous to the SPD and PLSD-T mutations so far detected; the specific site of these mutations seems to allow abnormal molecules to escape from mRNA decay. This evidence has led to the hypothesis that the combination of toxic effects arising from the accumulation of altered collagen chains inside the chondrocytes, diminished collagen fibril formation, and alteration of a putative signaling function of the carboxy-propeptide of type 2 collagen are the root cause of SPD and PLSD-T pathogenesis[9].

In conclusion this family is an excellent example of the need for a correct diagnosis in adulthood, the evidence of wide variability of these type of collagenopathies in terms of etiology and pathogenesis. Genetic counseling in these situations is very challenging and should take into account all the different aspects of rare disease.

## Competing interests

The authors declare that they have no competing interests.

## Authors' contributions

LT and BA are the two gynecologists who followed patient 1 from the 17^th ^to the 32^nd ^week of gestation. SG and EA performed the molecular analysis of COL2A1 gene. They described the method and results in the text. LC is the paediatrician who followed patient 2 during post-natal period. ASF is a paediatrician with a major expertise in skeletal dysplasia who contributed to the diagnosis of patient 1. MFB, VB, BG, FN and FL conceived and designed the work, reviewed the literature and the differential diagnosis, and delineated the critical point for the discussion. They described the general notion about SPD: clinical features, genetics (description, function and expression of the gene). All the authors gave their contribution to the drafting and critical review of the article. Each author participated actively to the discussion. The revised version was approved by all authors before re-submission. On the basis of these considerations, each author meets the criteria for authorship.

## Consent

Written informed consent was obtained from the patient for publication of this case report and accompanying images. A copy of the written consent is available for review by the Editor-in-Chief of this journal.
